# Analysis and validation of aging-related genes in prognosis and immune function of glioblastoma

**DOI:** 10.1186/s12920-023-01538-3

**Published:** 2023-05-19

**Authors:** Jianhua Mu, Jianan Gong, Miao Shi, Yinian Zhang

**Affiliations:** 1 The Second School of Clinical Medicine, Southern Medical University, Guangzhou, China; 2 School of Biomedical Engineering, Southern Medical University, Guangzhou, China; 3 Department of Neurosurgery, Zhujiang Hospital, Southern Medical University, Guangzhou, China

**Keywords:** Aging, GBM, TCGA, Bioinformatics, Prognosis

## Abstract

**Background:**

Glioblastoma (GBM) is a common malignant brain tumor with poor prognosis and high mortality. Numerous reports have identified the correlation between aging and the prognosis of patients with GBM. The purpose of this study was to establish a prognostic model for GBM patients based on aging-related gene (ARG) to help determine the prognosis of GBM patients.

**Methods:**

143 patients with GBM from The Cancer Genomic Atlas (TCGA), 218 patients with GBM from the Chinese Glioma Genomic Atlas (CGGA) of China and 50 patients from Gene Expression Omnibus (GEO) were included in the study. R software (V4.2.1) and bioinformatics statistical methods were used to develop prognostic models and study immune infiltration and mutation characteristics.

**Results:**

Thirteen genes were screened out and used to establish the prognostic model finally, and the risk scores of the prognostic model was an independent factor (*P* < 0.001), which indicated a good prediction ability. In addition, there are significant differences in immune infiltration and mutation characteristics between the two groups with high and low risk scores.

**Conclusion:**

The prognostic model of GBM patients based on ARGs can predict the prognosis of GBM patients. However, this signature requires further investigation and validation in larger cohort studies.

**Supplementary Information:**

The online version contains supplementary material available at 10.1186/s12920-023-01538-3.

## Introduction

Glioblastoma (GBM) is the most common malignant brain tumor in adults, accounting for 47.7% of all malignant tumors in the central nervous system [1]. The prognosis for GBM is poor, with a median survival of only 12 months after standard surgical resection and radiotherapy. Unfortunately, the addition of concomitant temozolomide-based chemotherapy has only improved the median survival to 14.6 months [2], suggesting that GBM has a high mortality rate. The age at diagnosis is an important risk factor for GBM, with the median age being 65 years [3]. Morbidity rate also increases with age, peaking at 75–84 years of age as reported [4]. Age impacts the prognosis of GBM as well [5, 6]. Researches conducted by the French Institute du Cancer (INCa) and other laboratories have demonstrated that the overall survival of patients with GBM decreases with the increase of age class of patients [5, 7]. These findings highlight the need for early detection and intervention in older patients. GBM with IDH-wild type is classified by the World Health Organization (WHO) as Grade IV malignancy due to its high invasiveness [8], which presents a significant threat to public health and emphasizes the importance of identifying specific targets related to GBM. Therefore, active search for new and effective treatments for GBM is urgently needed.

Although the correlation between age and the prognosis of glioblastoma has been established [5], the effect of age on the progression of glioma still remains unclear. Aging is characterized by senescence, which refers to the degeneration of the declined function of organs and tissues over time. Furthermore, senescence operates at both the molecular and cellular levels [9]. Cellular senescence describes the process in which cells stop proliferating irreversibly after a limited number of divisions [10]. This process has been shown to play a crucial role in cancer, where it may be initiated as a protective mechanism to suppress the uncontrolled growth of cancer cells [11, 12]. Aging-related genes (ARGs) regulate the process of cellular senescence and are highly associated with the development of various cancers [13, 14]. As for glioma, the role of cellular senescence in tumorigenesis is known to be dual, while its mechanism remains complex and not yet fully understood so far. Previous studies have indicated that glioblastoma in which EGFR signaling pathway is activated may inhibit cellular senescence by increasing the expression of *VEGFR2* and hence maintains its own invasiveness [15]. Another important factor is the senescence-associated secretory phenotype (SASP). For instance, astrocyte senescence and SASP induced by ionizing radiation (IR) facilitate the growth and migration of cancer cells through the secretion of SASP-associated factor HGF [16]. However, sodium butyrate-induced cellular senescence has been shown to occur with the inhibition of glioma cell invasion concurrently [17], suggesting that cellular senescence may also play a suppressive role in tumor development in certain cases. Similar results have been reported in skin tumors [18], pulmonary tumors [19], and breast tumors [20]. Therefore, aging-related genes may affect the occurrence and development of glioblastoma through complex signaling pathways. The function of aging-related genes needs to be better elucidated, as a fully understanding of it could provide a basis for developing new targets and approaches to clinical treatment of patients with GBM.

To gain a better understanding of how the genetic composition of cancer affects clinical prognosis, researchers have established comprehensive genome-wide gene expression sets, such as the Cancer Genome Atlas (TCGA), to classify and detect genomic abnormalities in large patient cohorts worldwide. Among these datasets, studies on aging-related genes and patient prognosis have been carried out across a variety of cancers with some significant progress having been achieved, including breast cancer, gastric cancer, ovarian cancer, and rectal cancer [21–24]. Recent bioinformatics-based screening has identified *CTSC* as a potential candidate since knock-down of *CTSC* has been found to be able to increase cell aging in glioma cell lines [25]. However, to the best of our knowledge, there have been few systematic studies on the relationship between aging-related genes and glioblastoma. Therefore, the establishment of prognosis-related models for glioblastoma based on aging-related genes could be helpful for better prediction of patient outcomes and may also contribute new insights into the pathogenesis of GBM.

## Materials and methods

### Data acquisition

The RNA-seq transcriptome data and clinical information for GBM patients were sourced from the Cancer Genomic Map TCGA database (https://portal.gdc.cancer.gov/) (n = 143), CGGA database (https://www.cgga.org.cn/) (n = 218) [26], and GEO database (https://www.ncbi.nlm.nih.gov/geo/) (GSE83300, n = 50). We obtained a list of 307 aging-related genes from the Human Ageing Genomic Resources [HAGR, Human Ageing Genomic Resources (senescence.info)] [27] (Additional file 1: Table S1). Patient data were subject to exclusion if any relevant information was missing to ensure the reliability and stability of the analysis. The remaining relevant data were processed using R software (v4.2.1).

### Cox regression analysis

To identify the aging-related genes (ARGs) most closely associated with overall survival of GBM patients, differential analysis of ARGs constructed by R package "DEseq2" was used to analyze differential gene expression between tumor and normal tissues in GBM patients. Differentially expressed genes (DEGs) were identified with the threshold determined to be |logFC|> 1 and *P* < 0.05. 116 ARGs obtained from TCGA data set. Results were displayed using forest plots created with the R packages "forestplot" and "survival". To explore potential protein–protein interactions, we utilized the STRING database [28] and Cytoscape software (v3.7.2) [29]. Data acquisition was performed using STRING, while data display was carried out using Cytoscape.

### Analysis of pathways and cellular functions of ARGs in GBM patients

We explored the enriched molecular mechanisms and cellular functions of the differentially expressed ARGs identified through differential analysis with Gene Ontology (GO) [30], Kyoto Encyclopedia of Genes and Genomics (KEGG) pathway analysis [31], and Gene Set Enrichment Analysis (GSEA) [32]. R package "ClusterProfiler" was applied to visualize the results of our analysis.

### Tumor mutation burden analysis

Somatic mutations presented in VarScan file format were downloaded from https://portal.gdc.cancer.gov/repository, while copy number variation files were curated from UCSC Xena online. For analysis of the tumor mutational burden (TMB), we employed the R package "maftools" [33], and the result was displayed using waterfall diagrams.

### Immune infiltration

To explore and quantify immune cell infiltration, we utilized the "CIBERSORT" package of R software. This package employs a validated deconvolution algorithm to characterize cell composition based on a white blood cell characteristic matrix (LM22) [34], which includes characterization of various immune cells, such as macrophages (M1 macrophages, M2 macrophages, and M0 macrophages), T cells (T follicular helper cells, resting memory CD4 T cells, activated memory CD4 T cells, γδ T cells, CD8 T cells, Tregs, and naïve CD4 T cells), resting natural killer (NK) cells, activated NK cells, resting mast cells, activated mast cells, memory B cells, resting dendritic cells (DC), activated DC, naïve B cells, monocytes, plasma cells, neutrophils, and eosinophils. We used CIBERSORT to quantify immunocyte infiltration in each sample and compared the results between different groups. Additionally, we calculated the stromal score, immune score, estimate score and tumor purity using gene expression data with the R package "estimate".

### Prognostic risk model and validation of aging-related genes

To further identify prognostic genes, we used Least Absolute Shrinkage and Selection Operator (LASSO) regression analysis via the R packages "glmnet" to identify the prognostic genes. The risk score for each patient was calculated using the following formula:

$$ Risk\;Score = \sum {Coef\left( i \right) \times Expr\left( i \right)} $$

*Coef(i)* represents the coefficient and *Expr(i)* represents the expression level of a particular gene. We utilized the "predict" function in the R package "glmnet" to calculate the risk score of individual patients. According to the median risk score, patients with GBM were then divided into high-risk and low-risk groups. At the same time, R software is used for model construction and verification. The train and test sets included 79 and 90 GBM patients, respectively, both sourced from the CGGA database after data processing. Subsequently, we utilized the R package "timeROC" to generate receiver operating characteristic (ROC) curves and plotted Kaplan–Meier (K-M) survival curves with package "survival" to demonstrate the results.

### Drug sensitivity analysis

To select potential drugs for the treatment of GBM patients, we utilized expression data and drug data downloaded from the CellMiner database (https://discover.nci.nih.gov/cellminer/home.do) [35] for drug sensitivity analyses. The National Cancer Institute (NCI) 60 data is a dataset with a total of 60 cancers and its cell lines were derived from nine different cancers. Valid data were screened out first and missing values were excluded from the expression data. Only FDA-approved drug data were selected for further analysis. Meanwhile, specific genes were selected for drug sensitivity prediction analysis after data filtering. To predict drug sensitivity based on specific genes, Pearson's correlation analysis was used to evaluate the relationship between the expression levels of these genes and drug response.

### Statistical analysis

All statistical analysis was conducted in the R statistical software (v4.2.1) using established procedures. Cox regression analysis was employed to identify prognostic genes, while Kaplan–Meier method was used to conduct survival analysis. The correlation between two continuous variables was examined by Spearman's correlation analysis. AUC was calculated to describe patient survival at 1–5 years and used to assess the predictive power of risk score. Significant differences in each LM22 fraction were compared by the Mann–Whitney U test. Additionally, all statistical tests were two-tailed, unless otherwise specified. Statistical significance was set at *P* < 0.05.

## Results

### Identification of differentially expressed ARGs

**Fig. 1:**
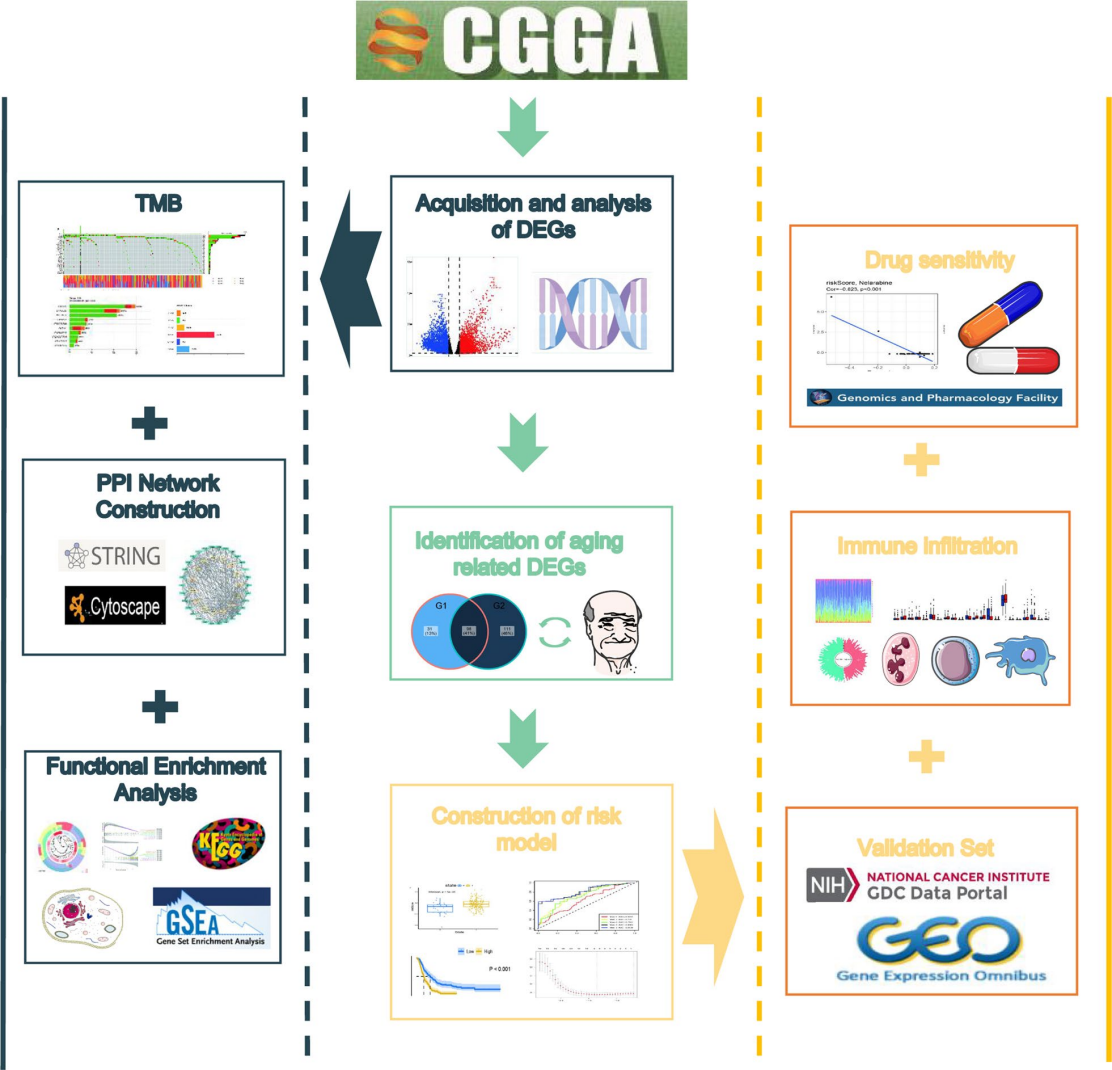
Flow of chart

**Fig. 2:**
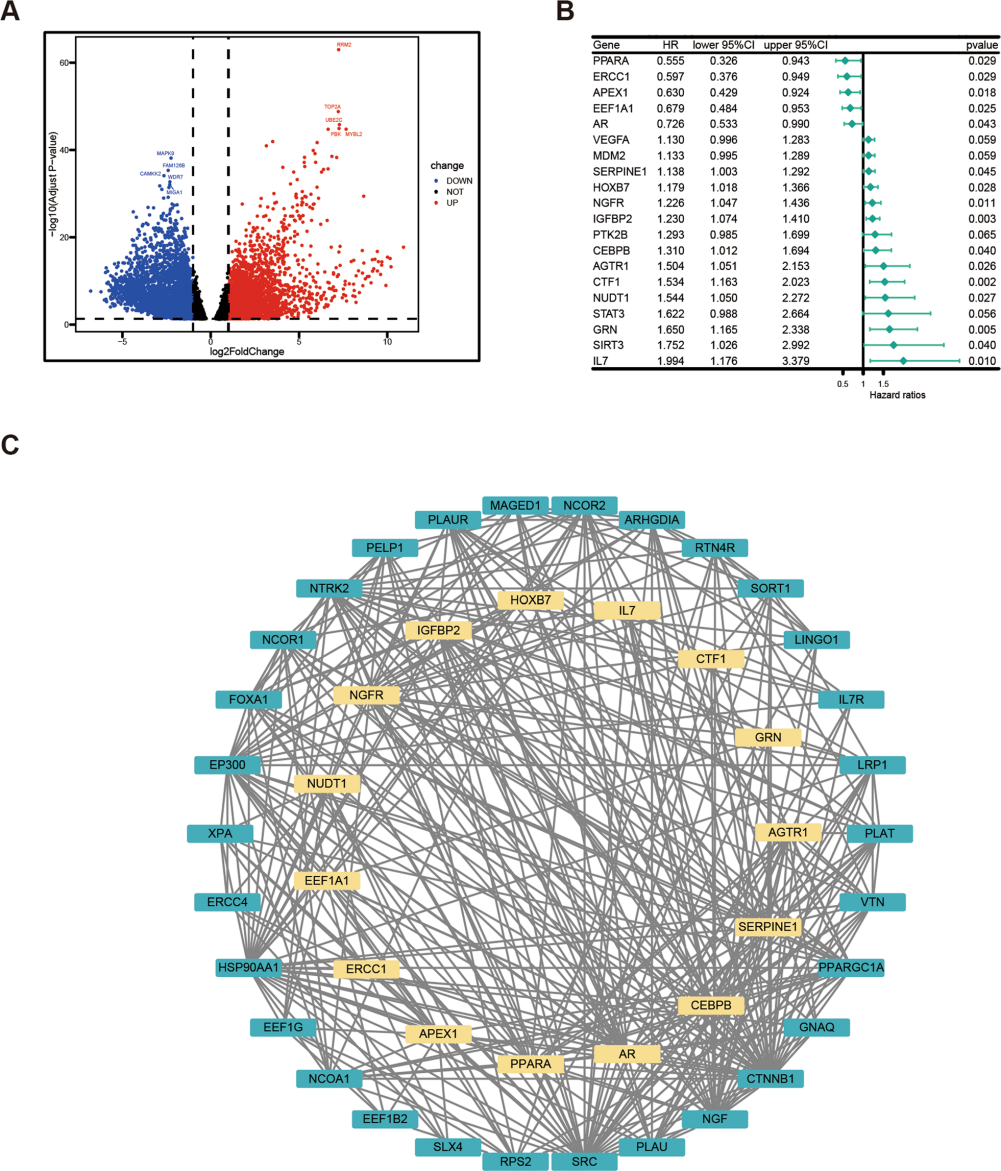
Analysis of DEGs. **A** Volcano map of DEG. **B** Forest map of differentially expressed ARGs using univariate Cox regression analysis. **C** Interaction network of ARGs from the STRING database

**Fig. 3:**
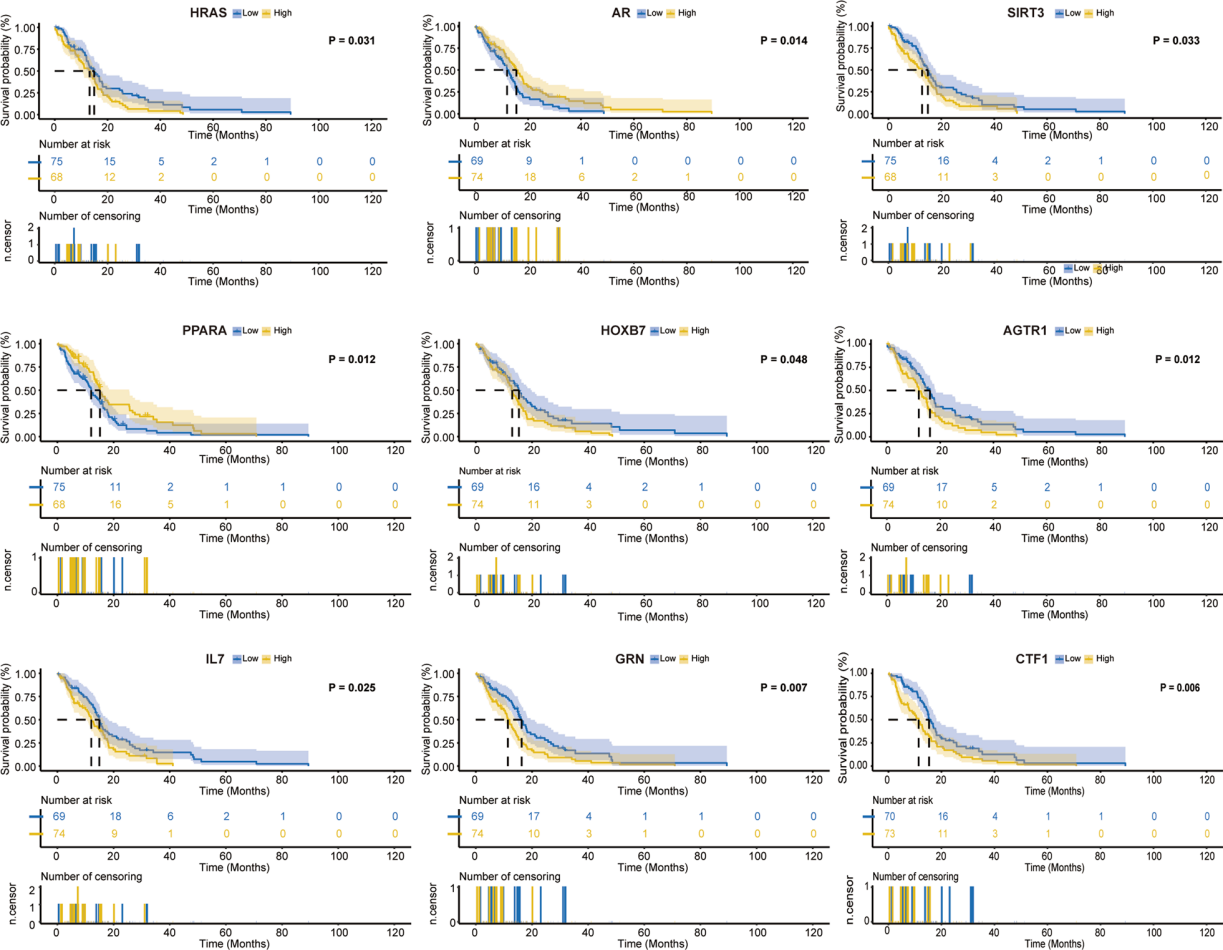
Kaplan–Meier survival curves of the first 9 genes most significantly correlated with prognosis

**Fig. 4:**
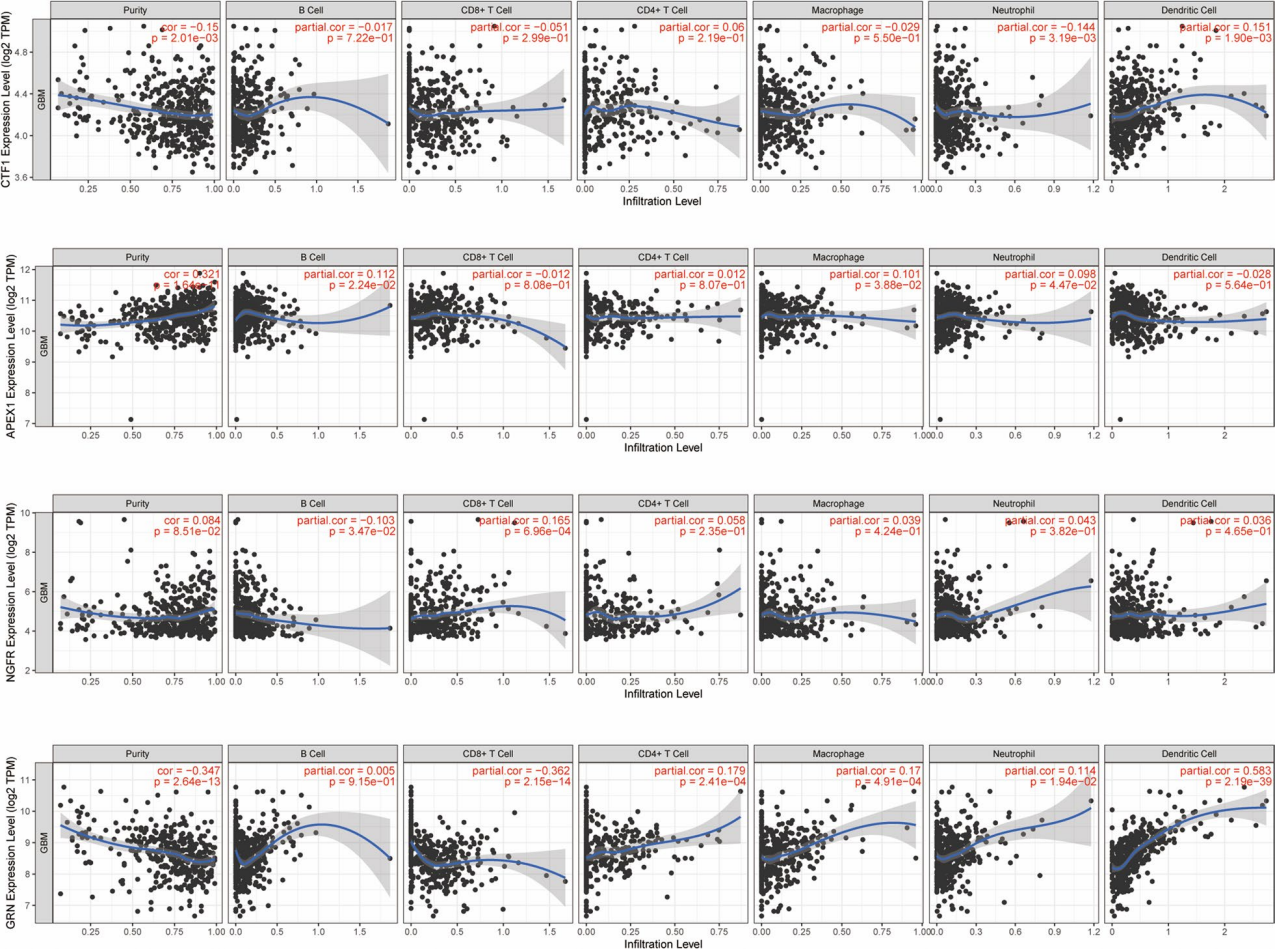
Correlation between the top 5 most significant ARGs and immunocyte infiltration

The workflow of this study is shown in Fig. 1. In this study, differentially expressed genes were analyzed using the “DESeq2” package of R software. Specifically, gene expression profiles and clinical information from 143 GBM patients, which were statistically significant according to the TCGA database, were utilized for the analysis. The results revealed that there was a significant difference in the expression of 116 genes out of 307 ARGs (Fig. 2A). Among them, 33 ARGs were down-regulated and 83 genes were up-regulated. Furthermore, univariate Cox regression analysis was performed on these 116 ARGs to examine the relationship between their expression and prognosis (Fig. 2B). The top 20 genes with optimal statistical significance were selected for further analysis. To further explore the relationships among these genes, a correlation network consisting of 15 genes was established using the STRING database and Cytoscape (Fig. 2C). Additionally, Kaplan–Meier survival curves were plotted for these 15 genes to examine their relationship with prognosis (Fig. 3). Moreover, the immune infiltrates for the five most significant genes were plotted using the Timer database (Fig. 4).

### Analysis of tumor mutation burden of differentially expressed ARGs

**Fig. 5:**
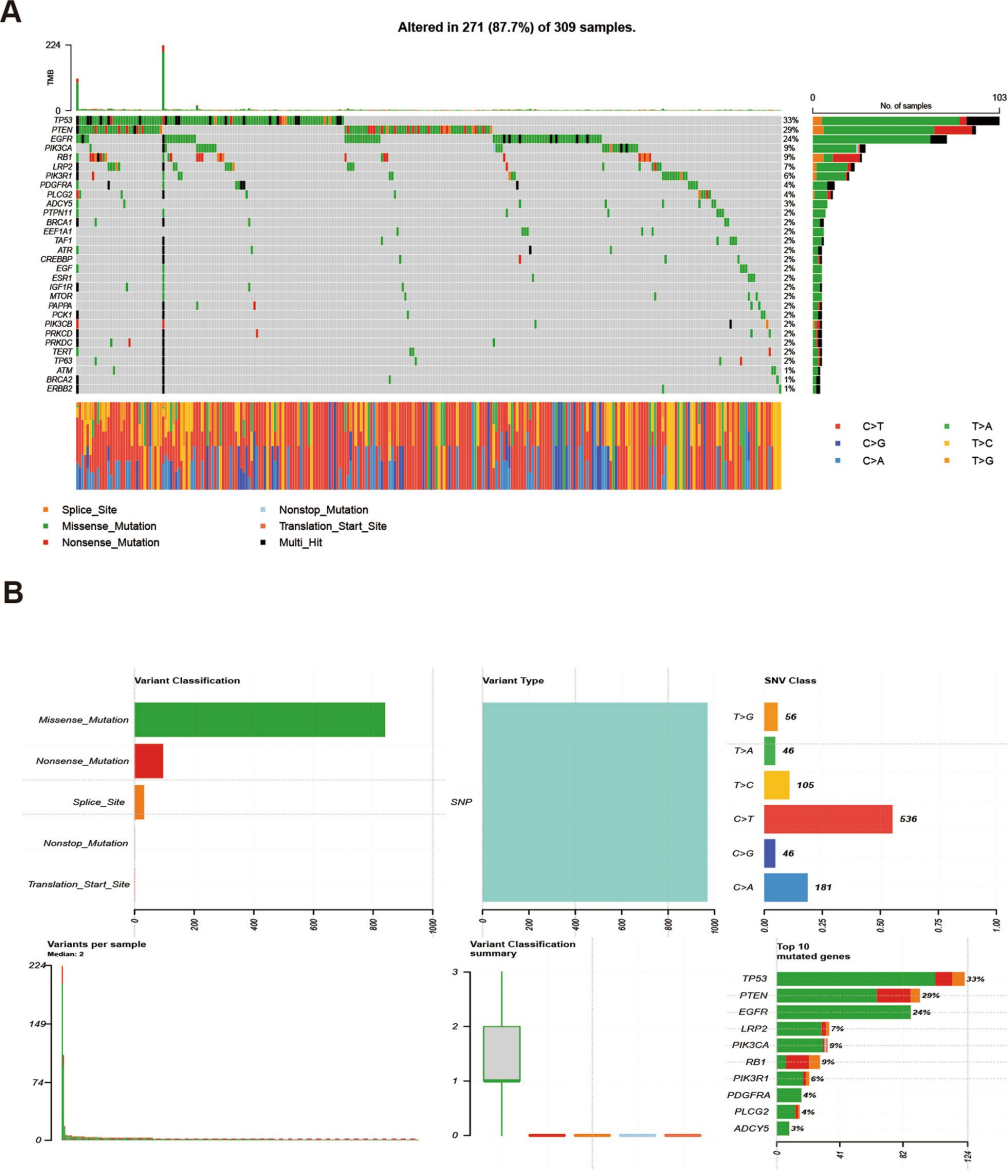
Overall results and waterfall plot of TMB of ARGs in GBM patients

To further investigate the genetic alterations of the 116 genes identified in our study, we conducted a tumor mutation burden analysis using the R package “maftools”. Our analysis revealed that missense mutations were the most common mutation classification and SNP was the most common mutation type (Fig. 5). Interestingly, the mononucleotide variation mainly occurred in the form of C > T.

### Analysis of functional enrichment

**Fig. 6:**
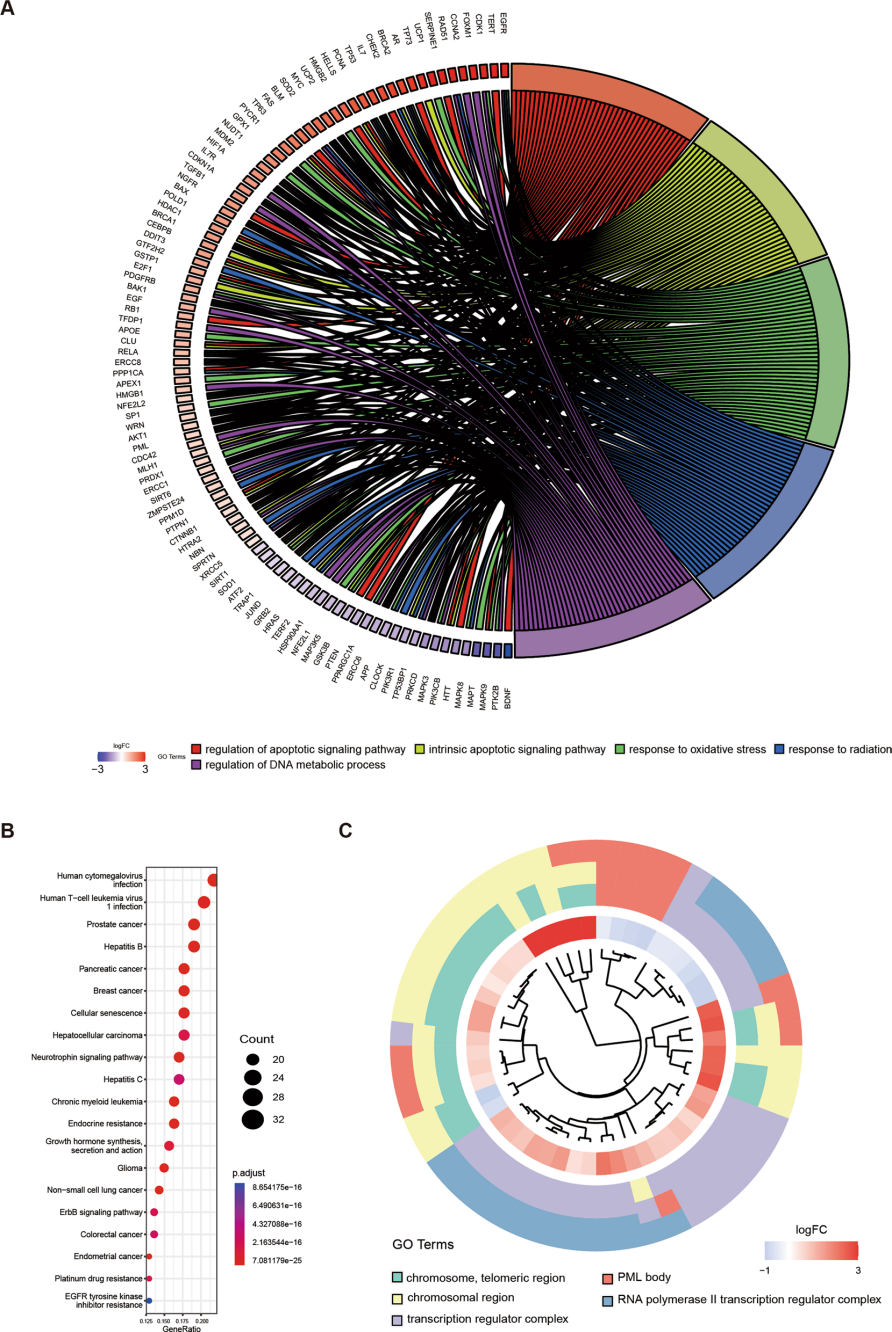
Results of enrichment analysis. **A** Biological processes enrichment of differential genes of GO. **B** Enrichment result of KEGG.** C** Cellular components enrichment of differential genes of GO

**Fig. 7:**
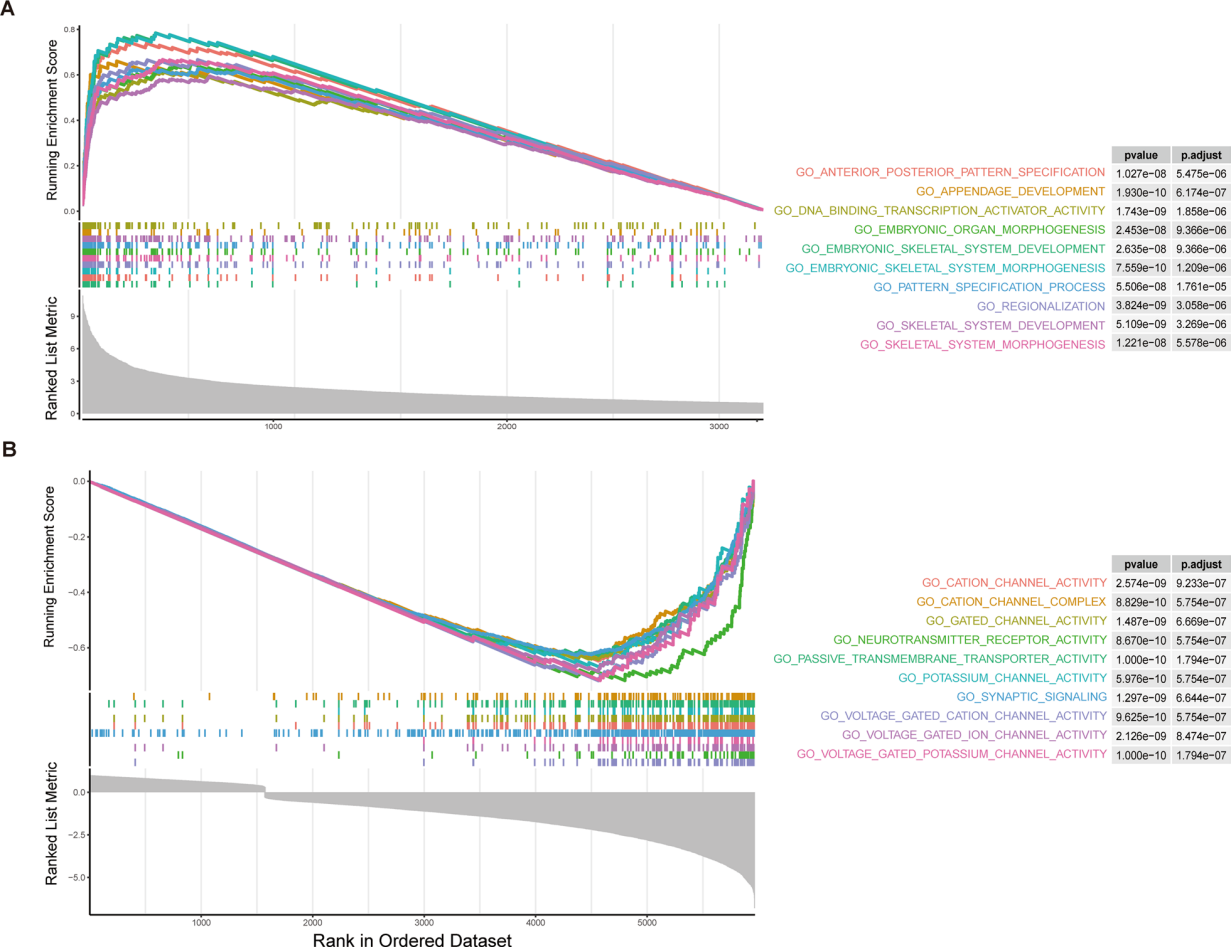
GSEA enrichment analysis. **A** GSEA enrichment result of up-regulated differential genes. **B** GSEA enrichment result of down-regulated differential genes

To elucidate the biological functions of the differentially expressed ARGs, we performed functional enrichment analysis using the GO and KEGG databases. GO enrichment analysis revealed that the differentially expressed ARGs were primarily enriched in five biological processes (Fig. 6A) and five cellular components (Fig. 6C), including regulation of apoptotic signaling pathways, intrinsic apoptotic signaling pathways, response to oxidative stress, response to radiation, regulation of DNA metabolic process, chromosomal region, and transcription regulator complex. Furthermore, KEGG enrichment analysis demonstrated that both upregulated and downregulated DEGs were involved in Neurotrophin signaling pathways as well as disease pathways such as Human cytomegalovirus infection, Human T-cell leukemia virus 1 infection, and Prostate cancer (Fig. 6B). To further investigate the functional roles of the differentially expressed ARGs, we performed gene set enrichment analysis (GSEA). Our results showed that the differentially up-regulated genes were mainly enriched in DNA binding transcription and Embryo organogenesis, while the differentially down-regulated genes were mainly enriched in Cation channel activity and Cation channel complex (Fig. 7).

### Immune infiltration

**Fig. 8:**
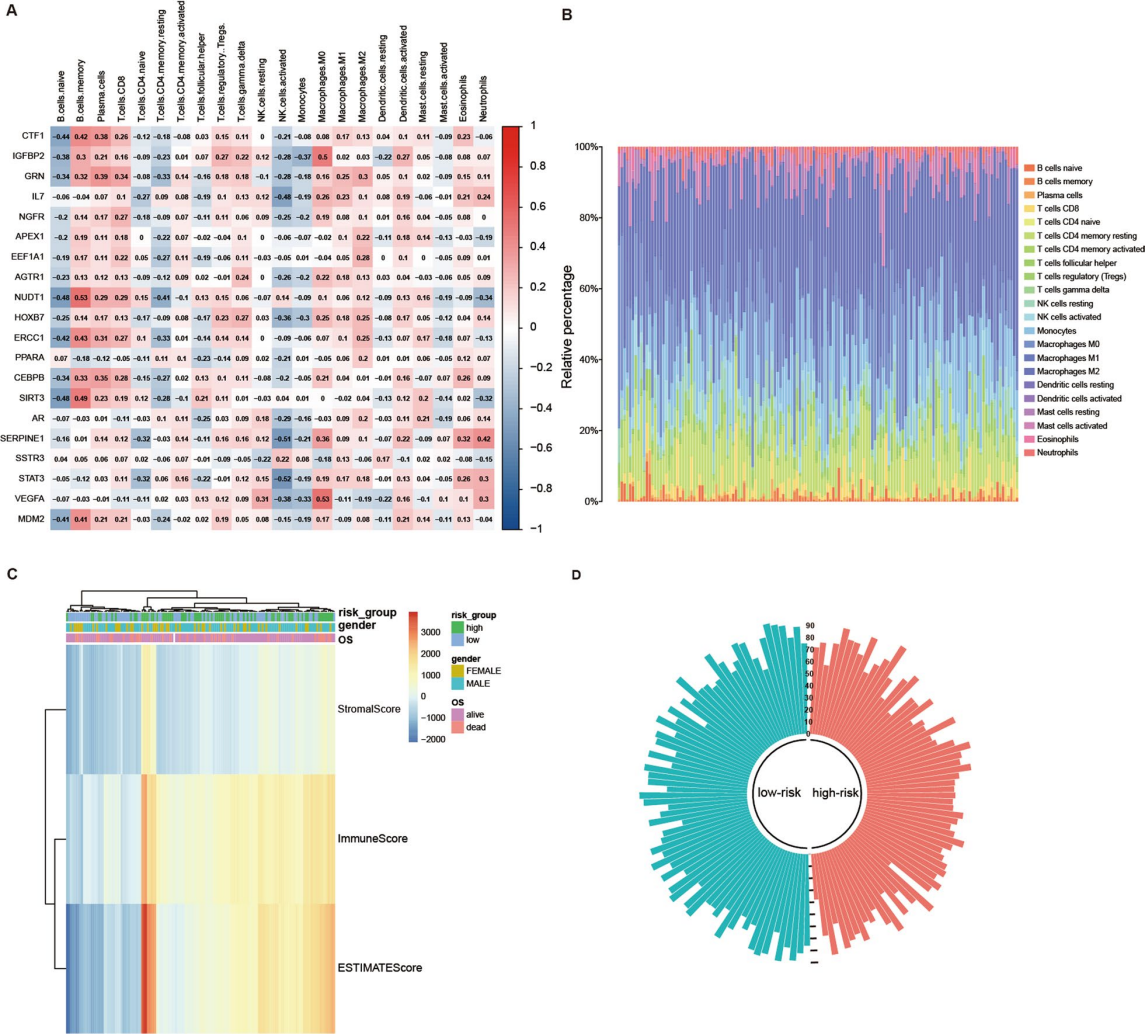
Analysis of immune infiltration in patients with GBM based on TCGA database. **A** Heat map of the correlation between 22 immune cells and ARGs. **B** The proportion of immune cell infiltration in 143 GBM patients. **C** Heatmap of the 3 immune scores and risk scores.** D** Circular histogram of purity score

The differential infiltration of immune cells in tumor tissues will help researchers understand the mechanism of tumor immune monitoring better. To investigate the immune infiltration level differences between high-risk and low-risk GBM patients, we utilized the “CIBERSORT” package to obtain immune cell profiles from 218 patients in the CGGA database (Fig. 8A, B). We further analyzed the correlation between 22 immune cells and identified *VEGFA* as positively correlated with Macrophages M0 (r = 0.53) and *STAT3* as negatively correlated with NK cells activated (r =  − 0.52). The “Stromal Score”, “Immune Score” and “Estimate Score" for 143 GBM patients were calculated using the “estimate” package, and the relationship between the three scores and the clinical information of patients was shown by a heat map (Fig. 8C). In addition, the circular histogram illustrated the tumor purity (Fig. 8D).

### Construction of the prognostic model with ARGs

**Fig. 9:**
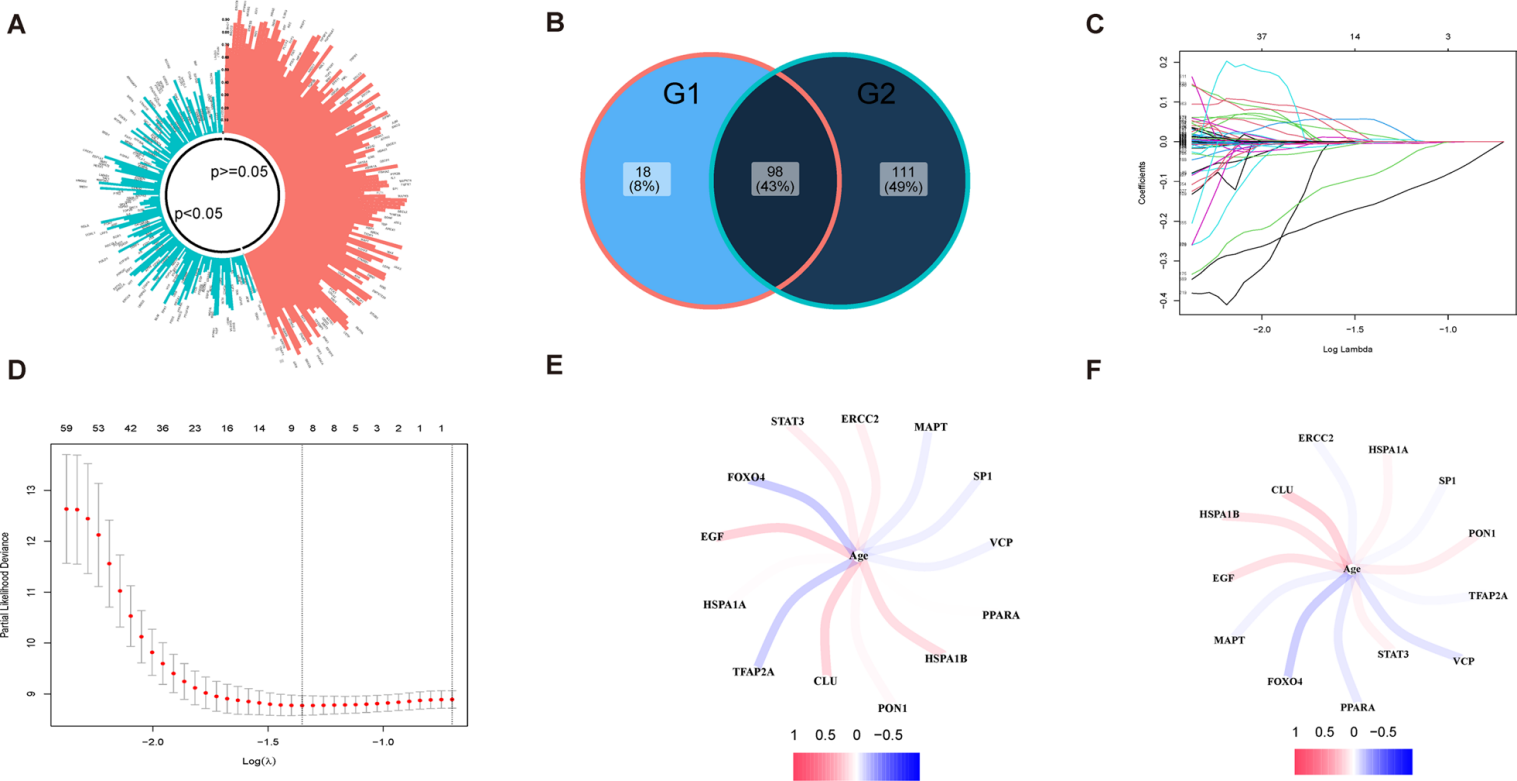
Construct prognosis model with candidate. **A** Circular histogram of P-value from Pearson correlation analysis. **B** Selection of candidate genes. G1:116 DE-ARGs. G2: 209 DEGs that significantly correlated with age. **C** LASSO coefficients of the 13 ARGs. **D** Identification of genes for construction of prognostic risk score model. **E–F** Correlation between Prognostic Genes and Age

**Fig. 10:**
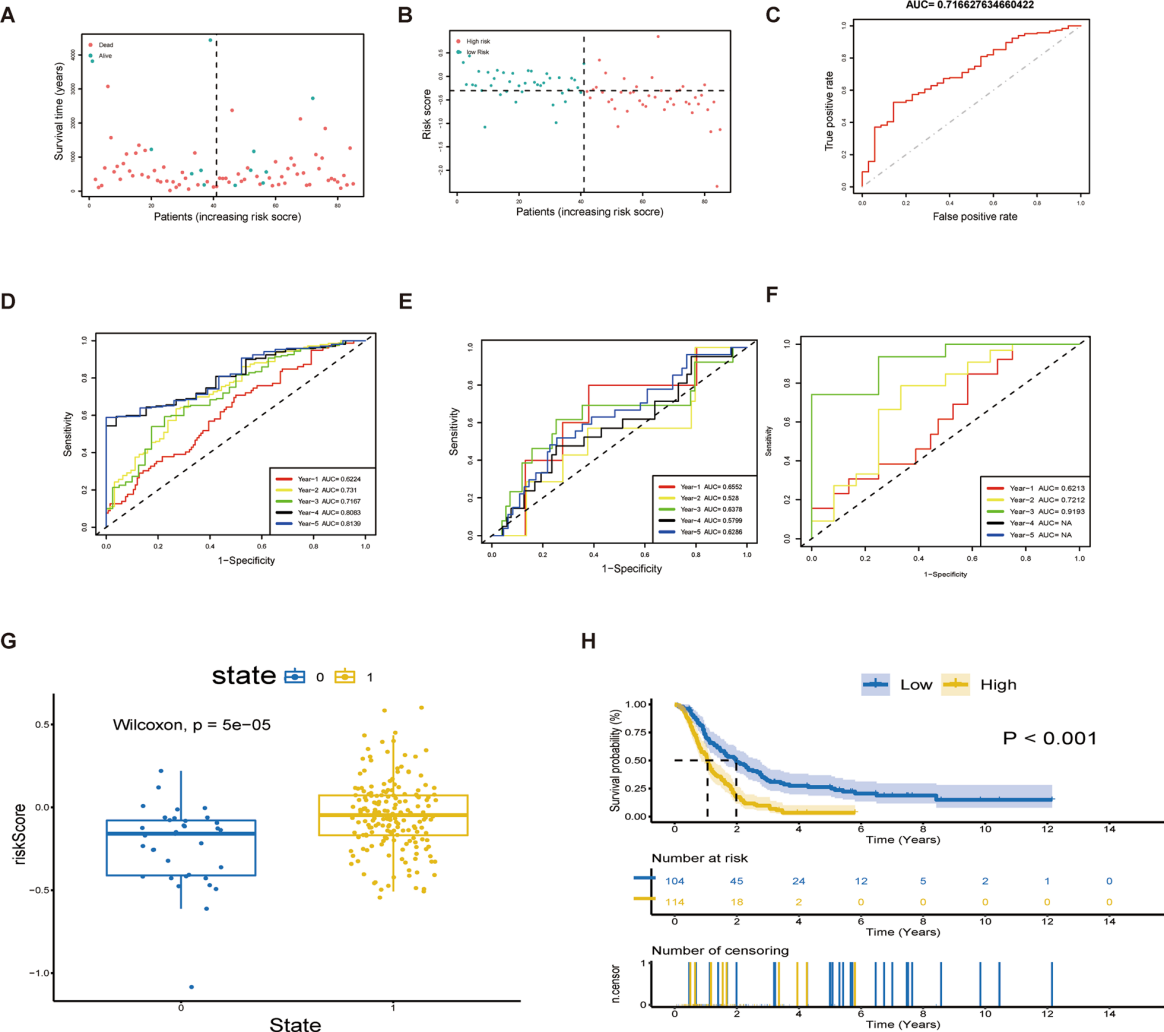
Performance of the prognosis model in the train set and test set. **A** Survival state distribution in train set. **B** Risk score distribution in train set.** C** ROC curve of OS in train set.** D** ROC curve of OS for 5 years in train set. **E–F** ROC curve of OS for 5 years in test set. **G** Box plot of risk scores for different prognoses.** H** Kaplan–Meier survival curve of high-risk group and low-risk group

To identify modules relevant to clinical features, we utilized the data of 218 GBM patients from the CGGA database as the train set, while the data of 143 GBM patients from TCGA and 50 GBM patients from GEO as test set. By analyzing the differentially expressed genes (DEGs) between GBM tissues and normal tissues, we identified 209 DEGs that significantly correlated with age. We then intersected them with the 116 differentially expressed ARGs obtained previously, resulting in 98 candidate ARGs for the construction of the prognostic model (Fig. 9A, B). LASSO analysis further identified 13 crucial ARGs for constructing the prognostic model (Fig. 9C, D). Of these, *STAT3, EGF, VCP, HSPA1A, HSPA1B, SP1, TFAP2A*, and *CLU* showed positive correlations with the risk score, while *ERCC2, PPARA, PON1, FOXO4*, and *MAPT* displayed negative correlations with the risk score. The coefficients of the calculation formula for the risk score are presented in a table (Additional file 2: Table S2). The correlation between these prognostic genes and patients' age in both the train and test sets was shown (Fig. 9E, F). Based on the risk score calculation formula, we obtained the corresponding risk scores of GBM patients. The overall survival (OS) of patients with low-risk scores was significantly better than those with high-risk scores (Fig. 10A). Furthermore, the model demonstrated significant differences in prognosis and risk scores of patients with GBM (Fig. 10B). We assessed the fitting effect of the model in the train set using a ROC curve (Fig. 10C) and the results showed that the AUC values over five years were 0.622, 0.731, 0.717, 0.808, and 0.814, respectively (Fig. 10D). To further validate the performance of the ARG signature in predicting prognosis, we calculated the risk scores of the validation set and grouped them according to the score level using the same formula. The ROC curve showed the model's prediction effect in the test set (Fig. 10E, F).

### Characteristics of high-risk group and low-risk group

**Fig. 11:**
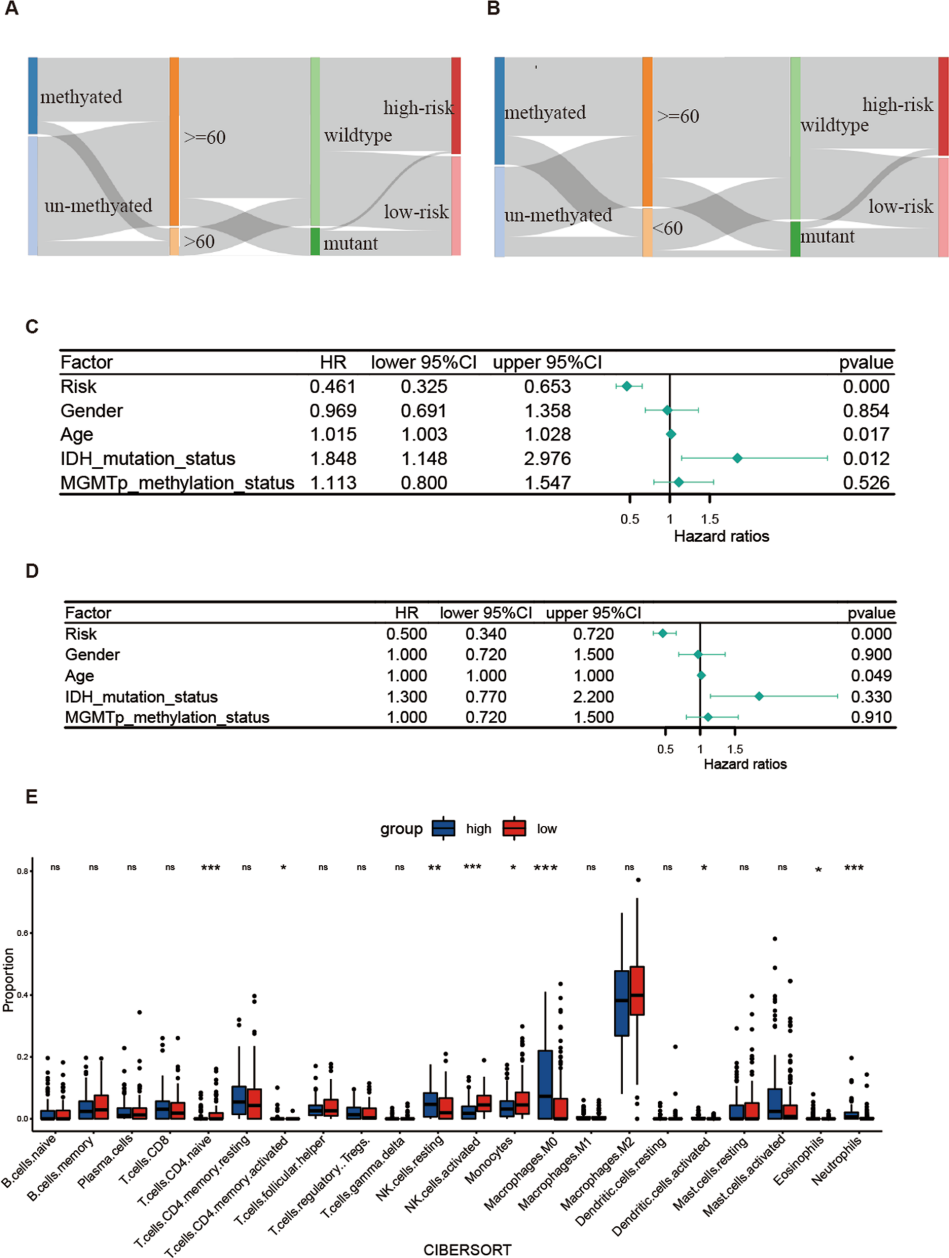
Mulberry figure of association between Clinical phenotype and forest plot for Cox regression. **A** Train set. **B** Test set. **C** Univariate Cox regression. **D** Multivariate Cox regression. **E** Comparison of immune cell infiltration between high-risk and low-risk group

To further examine the characteristics of the high-risk and low-risk groups, we analyzed the Kaplan–Meier (K-M) curve and risk score distribution of different survival outcomes. Significant differences were observed between these groups (Fig. 10G, H). In addition, the mulberry map displayed the relationship between the risk score subgroups and the clinical data of GBM patients (Fig. 11A, B). Our forest plots showed that the risk score was the most significant factor associated with patient's overall survival (Fig. 11C, D). However, it was inconsistent with the results of survival analysis, and we suspected that it was related to the amount of data. Moreover, comparisons between the high-risk and low-risk groups revealed that the high-risk population had significantly higher distributions of NK cells resting and neutrophils and significantly lower distributions of NK cells activated, monocytes, and T cells CD4 naïve (Fig. 11E).

### Correlation between risk score and drug sensitivity to chemotherapy

**Fig. 12:**
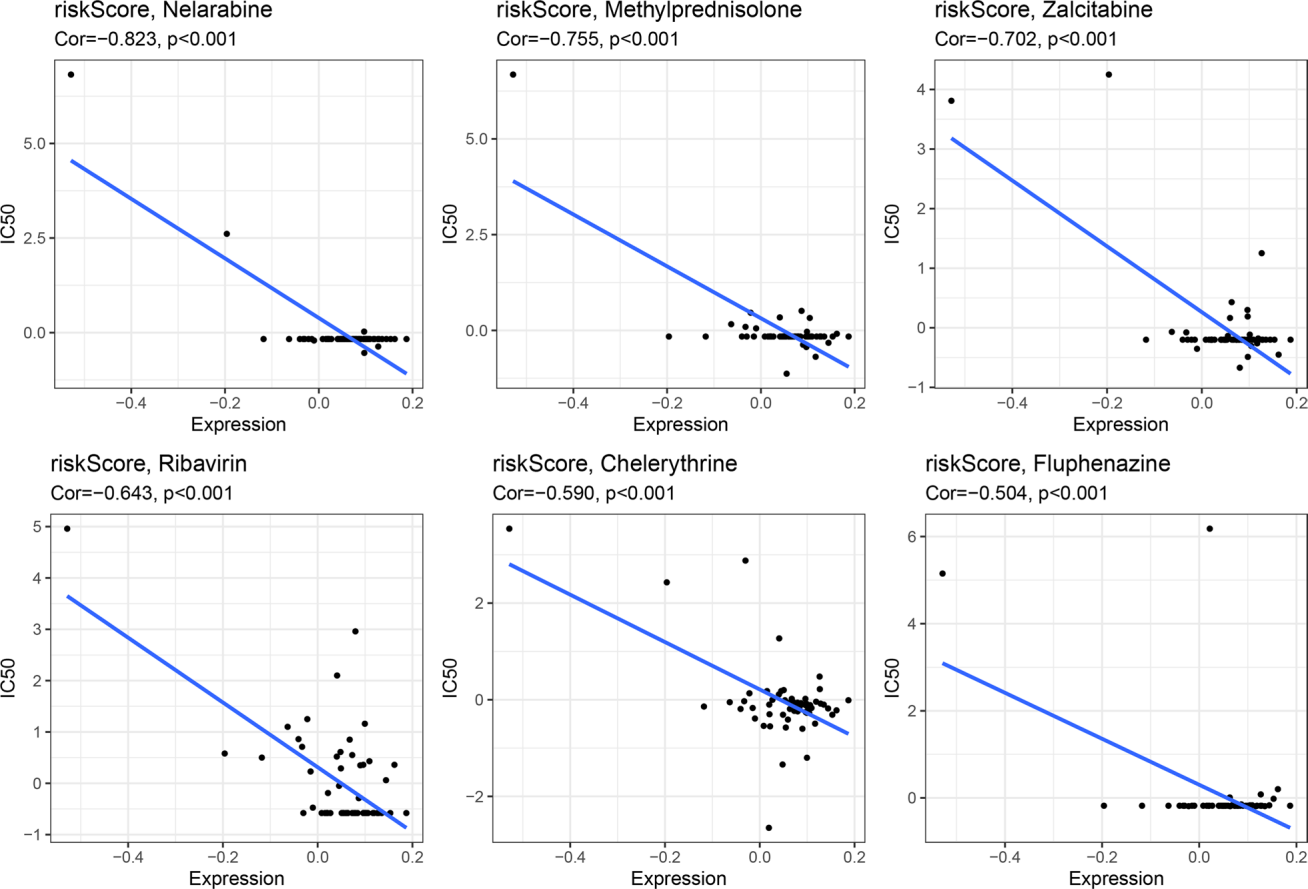
Association between these signatures and estimated IC50 value of drugs

To further examine the correlation between the characteristics of the NCI-60 cell line and drug susceptibility, Pearson's correlation analysis was conducted. Our analysis utilized risk scores and data from the Cellminer database, which allowed us to investigate the relationship between risk scores and drug susceptibility. We found a significant negative correlation between risk scores and Nelarabine, Methylprednisolone, Zalcitabine, Ribavirin, Chelerythrine, and Fluphenazine (*P* < 0.001). Moreover, we also analyzed the correlation between ARGs and drugs. Our results revealed a positive correlation between *CH25H* and Caffeic acid, as well as a negative correlation between *RAB37* and Nelarabine, Methylprednisolone, Zalcitabine, and Ribavirin (Fig. 12).

## Discussion

Cellular senescence is a crucial process in which the body eliminates unwanted cells and mediates tissue remodeling. This process is summarized by researchers as the senescence-clearing-regeneration model, which aims to promote cell metabolism initially [36]. However, in certain circumstances, aging cells may gradually accumulate and won't be replaced completely by new cells over time, potentially contributing to disease resulting from cellular senescence. It is widely acknowledged that cellular senescence plays a dual role in cancer development, either promoting or inhibiting cancer progression under specific conditions [37, 38]. Additionally, most studies on GBM have revealed that cellular senescence may contribute to its development [39–41], and may ultimately impact the process of tumor recurrence after radiotherapy and chemotherapy [16, 42]. Therefore, cellular senescence may act as a therapeutic resistance factor in GBM. In summary, understanding the role of cellular senescence in health and disease is essential for developing effective interventions and treatments.

In our study, gene expression data of GBM tissues and normal tissues acquired from the database were used to screen differential genes, and the obtained DEGs and ARGs were combined to get the differential expression genes of ARG. Univariate Cox regression analysis was performed using clinical data from the same database to identify the most prognostic differentially expressed ARGs. Bioinformatics statistical methods such as immune infiltration, Gene Set Enrichment Analysis (GSEA), Tumor Mutation Burden (TMB), and co-expressed genes were employed to demonstrate the biological function of these ARGs. To further validate the role of the identified ARGs in predicting the prognosis of patients with GBM, a risk scoring signature was constructed based on gene expression LASSO analysis. This approach helped in analyzing and confirming the role of the risk scoring model in predicting the prognosis of patients with GBM. Additionally, we also analyzed potential anticancer drug targets in patients with GBM.

We established a 13-aging-related-gene risk signature to predict the prognosis of glioblastoma (GBM) patients. Our results showed that patients classified as low-risk had a higher five-year survival rate of GBM, while those identified as high-risk had a poor prognosis. The risk signature included *STAT3, EGF, VCP, HSPA1A, HSPA1B, SP1, TFAP2A, CLU, ERCC2, PPARA, PON1, FOXO4,* and *MAPT*. The first eight genes were related to high risk while the latter five indicated low-risk. *STAT3* is a cytoplasmic transcription factor whose involved gene network plays a critical role in the progression and epithelial-mesenchymal transition (EMT) of GBM cells [43, 44]. Furthermore, *EGF* increases the expression of Netrin-4 in the U251MG cell line and prevents tumor cell senescence induced by DNA damage in GBM, hence it is regarded as a protective factor [45]. Researchers have also found that high levels of HDAC6 and low levels of p97/*VCP* may be responsible for resistance to TMZ treatment and endoplasmic reticulum (ER) stress in GBM cells [46]. *HSPA1A,* which is regulated by lncRNA NONHSAT079852.2, overexpresses in primary GBM cells, and is associated with the progression and recurrence of GBM [47, 48]. Moreover, studies have shown that *HSPA1B* inhibits apoptosis via the JNK pathway and is linked to the sensitivity of GBM cells to erlotinib [49]. SP1 acts as an activator of DLEU1 transcription and promotes the proliferation of GBM [50]. However, the roles of *TFAP2A* and *CLU* in GBM are yet to be fully elucidated. *TFAP2A* is the upstream transcription factor of *ITPKA*, which promotes the occurrence and development of lung adenocarcinoma (LUAD) by interacting with Drebrin1 [51]. Furthermore, clusterin expressed by *CLU* is a highly evolved and conserved glycoprotein that associates with the development of prostate, breast, pancreatic and many other cancers as reported [52]. The latter five genes were associated with a low-risk score. *ERCC2* is involved in nucleotide excision repair (NER), which may be related to the repair of DNA damage in glioma cells [53]. Researchers found that the odds ratio (OR) for glioma increases significantly in the population carrying homozygous variants of ERCC2 K751Q (QQ) [54]. In addition, *PPARA* is overexpressed in primary GBM and is associated with a favorable prognosis [55]. Studies have found that the serum level of *PON1* in glioma patients is lower than that of normal people [56]. Moreover, the PON enzyme coded by *PON1* could detoxify liquid peroxidation, suggesting that *PON1* may be the resistance factor of glioma [57]. *FOXO4* is down-regulated in GBM while its overexpression promotes apoptosis and inhibits the migration and invasion of cancer cells [58]. A study revealed that the *MAPT* gene-expressed Tau is a microtubule-associated protein and its restrained expression suppresses the growth and proliferation of GBM cells [59]. All 13 ARGs were involved in the pathogenesis of the disease. In conclusion, these results indicated that the 13 markers had potential clinical application in the future.

In our study, we observed that the differentially expressed genes were mainly enriched in apoptotic signaling pathways, oxidative stress response, and DNA metabolism based on GO enrichment analysis. Apoptosis is a key concept in tumor treatment, and studies have indicated that andrographolide can participate in the inhibition of the DBTRG-05MG cell line through the apoptotic signaling pathway [60], demonstrating its potential therapeutic effect on this disease. Furthermore, the inhibition of apoptotic pathways can significantly reduce the efficacy of anti-tumor therapy. For instance, in ovarian cancer, the inhibitory effect of inositol‐required enzyme 1α (IRE1α) on the apoptotic pathway resulted in poor clinical efficacy of AZD1775 [61]. Besides, malignant cells are known to have higher levels of intrinsic reactive oxygen species (ROS) compared to normal cells [62]. To maintain redox balance and survival, these cells activate their antioxidant defense systems and fight against the intrinsic oxidative stress [63]. Combination of AF and cold plasma has been reported to enhance the oxidative stress process to achieve the purpose of GBM treatment [64]. However, there is a scarcity of research on DNA metabolism and its role in the pathogenesis of GBM. Therefore, further research is needed to investigate the role of DNA metabolism and to verify its contribution to the pathogenesis of GBM.

The tumor microenvironment (TME) is known to be complex and diverse in its immune state [65]. Therefore, predicting response to immune checkpoint inhibitors (ICI) based on TME cell infiltration is an important procedure for improving the current efficacy of ICI and developing new immunotherapeutic regimens [66]. In our study, we analyzed immune infiltration and found significant differences in five immune cell infiltration degrees between high-risk and low-risk GBM patients, including NK cells resting, neutrophils, NK cells activated, monocytes, and T cells CD4 naïve. Several studies have reported treatment of GBM based on these immune checkpoints. For instance, the calcipotriol/TSLP/CD4 + T axis has been shown to activate CD8 + T and NK cells as a novel therapeutic modality [67]. Therefore, screening patients with GBM who have a high-risk score can help select an appropriate treatment strategy based on immune checkpoint inhibitors.

The analysis of immune infiltration revealed varying degrees of infiltration by T cells CD4 naïve, NK cells, Macrophages M0, and neutrophils in patients with different risk scores. Notably, CD4 + helper T cells have the ability to fully support the potential of CD8 + T cells in vivo [68], and support a lasting tumor-specific cytotoxic T cell response by guiding down-regulation of co-inhibitory receptors and enhancing CD8 + T cells' ability to infiltrate tumors [69]. Additionally, studies have indicated that calmodulin-dependent kinase kinase 2 (CaMKK2) reduces the amplification of effector CD4 + T cells to limit the tumor penetrance of GBM patients [70]. NK cells are also essential components of the immune system that play crucial roles in controlling microbial infection and tumor progression [71]. Furthermore, NK cells have been found to control the growth and metastasis of transplantable tumors in mouse models that NK cells are depleted through antibodies [72]. Presently, immunotherapy based on NK cells has been used to treat GBM. For example, FDA approved the use of allogenic NK cells derived from human placental hematopoietic stem cells for GBM therapy [73]. Additionally, CD73, an immune checkpoint found in tumor-infiltrating NK cells, has been discovered to be able to bind tumor cells, indicating its potential as a therapeutic target [74]. Neutrophils also play significant roles in tumor development. Tumor matrix infiltration causes tumor cells to undergo ferroptosis [75], while, in neuroinflammation, neutrophils can infiltrate the central nervous system. There is also a correlation between an increase in neutrophils and the severity of central nervous system diseases [76]. Our study results are consistent with past findings, indicating that GBM tumor infiltration reduces the enrichment of neutrophils [76]. Nevertheless, neutrophils have two sides to GBM. A study has shown that neutrophils have both anti-tumor effects in the early stages and tumor-promoting effects in the later stages [77]. Unfortunately, targeting neutrophils in GBM therapy remains in its infancy, partly due to its small numbers and unknown function.

In the drug sensitivity analysis, two ARGs, *RAB37* and *CH25H*, were found to be significantly correlated with multiple drugs. Specifically, the transcription and translation of *CH25H* have been found to increase in response to TNFα and IL1β in glioblastoma cell lines. Furthermore, the U87MG and GM133 GBM cell lines upregulate the synthesis and secretion of 25-hydroxycholesterol (25-OHC) to levels comparable to mouse macrophages derived from bone marrow under inflammatory conditions [78]. The anti-cancer activity of the designed CAPE analogues on GBM cells also demonstrates the proposed compounds' ability to interact with key residues [79]. Moreover, Yang et al. [80] found that *RAB37* mediates the secretion of *CHI3L1* in immune cells, highlighting that nCHI3L1 Abs have the potential to target cancer cells and the tumor microenvironment simultaneously. Ribavirin, a nucleic acid analogue, has been used as an antiviral agent against RNA and DNA viruses. Interestingly, analysis by FACS shows that ribavirin treatment, downstream of the p53 pathway, could induce apoptosis, indicating that both exogenous and endogenous apoptosis in malignant glioma cell lines is activated [81]. With no reports currently available on the treatment of GBM using other drugs, our findings provide a new direction for chemotherapy approaches.

We established a model to predict the prognosis of patients with GBM. However, this study has certain limitations that must be considered. Firstly, as a bioinformatics study, it relies on data from multiple historical data sets, and the number of samples included is relatively small. Therefore, in order to develop more reliable clinical applications, it is necessary to obtain prospective data from clinical cohorts to validate the results. Secondly, functional research investigations and animal experiments are necessary to verify the predictive accuracy of risk models and achieve a better understanding of the aging-related processes. These studies will help to identify possible mechanisms underlying the disease and facilitate the development of effective treatment strategies.

## Conclusion

This study predicts the prognosis of GBM patients using 13 ARGs. The risk score was found to be significantly associated with GBM prognosis, suggesting that this prognostic model may serve as an effective tool for predicting the prognosis of patients with GBM. However, further investigation and validation of this model is necessary, particularly in larger cohort studies, to ensure its reliability and generalizability to diverse patient populations.

## Supplementary Information

**Additional file 1. Table S1:** 307 Aging-related genes.

**Additional file 2. Table S2:** The coefficients of the calculation formula for the risk score.

## Data Availability

This study analyzed publicly available data sets. These data can be found here: Aging related genes can be downloaded from HAGR [Human Ageing Genomic Resources (senescence.info)]. RNA-seq and clinical data can be derived from TCGA (https://portal.gdc.cancer.gov/), CGGA (https://www.cgga.org.cn/) and GEO (https://www.ncbi.nlm.nih.gov/geo/query/acc.cgi?acc=GSE83300) database. Immunization, estimation, and matrix scoring data for TCGA-GBM samples were obtained from the ESTIMATE database (https://bioinformatics.mdanderson.org/estimate). Drug sensitivity data can be obtained from CellMiner (https://discover.nci.nih.gov/cellminer). The codes used in our study are available on reasonable request from the author of the communication.

## Abbreviations

GBM: Glioblastoma
ARG: Aging-related gene
TCGA: The Cancer Genome Atlas
CGGA: Chinese Glioma Genome Atlas
DEG: Differentially expressed gene
KEGG: Kyoto Encyclopedia of Genes and Genomes
CIBERSORT: Cell type identified by estimating relative subsets of RNA transcripts
GSEA: Gene set enrichment analysis
NCI: National Cancer Institute
LASSO: Least absolute shrinkage and selection operator
KM: Kaplan–Meier
ROC: Receiver operating characteristic
AUC: Area under the curve
OS: Overall survival
PCA: Principal component analysis
DNA: Deoxyribonucleic acid
